# Investigating the in-vitro bioactivity, biodegradability and drug release behavior of the newly developed PES/HA/WS biocompatible nanocomposites as bone graft substitute

**DOI:** 10.1038/s41598-024-61586-2

**Published:** 2024-05-11

**Authors:** Esmaeil Salimi, Mulazim Hussain Asim, Muhammad Nidzhom Zainol Abidin

**Affiliations:** 1 Faculty of Chemical and Materials Engineering, Shahrood University of Technology, Shahrood, 3619995161 Iran; 2 College of Pharmacy, University of Sargodha, Sargodha, 40100 Pakistan; 3 Department of Chemistry, Faculty of Science, Universiti Malaya, Jalan Profesor Diraja Ungku Aziz, 50603 Kuala Lumpur, Malaysia

**Keywords:** Bioactive film, Hydroxyapatite, Drug delivery, Bone tissue engineering, Biomedical materials, Biomedical engineering

## Abstract

The nucleation of carbonate-containing apatite on the biomaterials surface is regarded as a significant stage in bone healing process. In this regard, composites contained hydroxyapatite (Ca_10_(PO_4_)_6_(OH)_2_, HA), wollastonite (CaSiO_3_, WS) and polyethersulfone (PES) were synthesized via a simple solvent casting technique. The in-vitro bioactivity of the prepared composite films with different weight ratios of HA and WS was studied by placing the samples in the simulated body fluid (SBF) for 21 days. The results indicated that the the surface of composites containing 2 wt% HA and 4 wt% WS was completely covered by a thick bone-like apatite layer, which was characterized by Grazing incidence X-ray diffraction, attenuated total reflectance–Fourier transform infrared spectrometer, field emission electron microscopy and energy dispersive X-ray analyzer (EDX). The degradation study of the samples showed that the concentration of inorganic particles could not influence the degradability of the polymeric matrix, where all samples expressed similar dexamethasone (DEX) release behavior. Moreover, the in-vitro cytotoxicity results indicated the significant cyto-compatibility of all specimens. Therefore, these findings revealed that the prepared composite films composed of PES, HA, WS and DEX could be regarded as promising bioactive candidates with low degradation rate for bone tissue engineering applications.

## Introduction

Conventionally, autogenous grafts have been utilized to repair bone defects generated by infection, trauma or tumor. But, immunogenic issues and limited accessibility have obliged the orthopedician to employ artificial bioactive materials as reliable alternatives^1^. It is well known that the bioactive materials are able to chemically interact with the surrounding living bone via establishment of an apatite interface layer. CaO–SiO_2_ containing compounds such as bioactive glasses have been evaluated as bioactive substances in bone tissue engineering^2,3^. Wollastonite (CaSiO_3_) is a biodegradable and bioactive CaO–SiO_2_-based glass that has been extensively investigated as a potential bone filler or drug carrier^4,5^. A bone-like apatite layer forms on the wollastonite immediately upon placing in the simulated body fluid (SBF)^6,7^, cell culture medium^8^, or grafting in vivo^9^, which effectively take part in the establishment of strong bonds between the bioactive compounds and the adjacent bones, where the non-bioactive materials suffer the lack of such layer^10^. Salmani et al.^11^ have prepared three-dimensional scaffolds using bioactive wollastonite, bioglass and magnetic nanoparticles (MNP). Interestingly, the quantity of magnetic nanopaeticles has affected the biological and mechanical properties of the scaffold, where the sample with 10 wt% MNPs has presented a better apatite formation on porous scaffolds after 28 days in SBF solution and also the sample with 5 wt% MNPs has been almost completely dissolved due to its insufficient strength and weak chemical activity compared with other sample. It has been proven that compared to the biocompatible glasses, glass ceramics and calcium phosphate materials, the apatite layer forms with a higher rate on the wollastonite surface, which is related to the silicate group that contribute in the metabolic activities when the bone is being generated^12–14^. Nevertheless, the brittle nature and poor chemical stability of wollastonite has limited its applications.

Hydroxyapatite (HA) as an excellent biocompatible and osteoconductive ceramic has attracted much attention, recently. Although, the moderate degradation rate of the HA has inhibited the complete bone replacement and consequently has limited its clinical usage^15,16^. In order to solve this problem, HA has been combined with other bioceramics possessing higher degradation rate. Preparation of composite materials is considered as an effective approach to overcome the drawbacks of each individual component. Bioactive glasses have been employed to enhance the mechanical strength, and bioresorption rate of the HA bioceramics^17,18^. Among a variety of bioglasses, WS has been broadly applied in bone tissue engineering to develop mechanically stable hybrids^19,20^. Sprio et al.^21^ prepared bone scaffolds based on the HAp/Ca_2_SiO_4_ composites. Kokubo^22^ fabricated bioactive glass–ceramic composed of wollastonite and apatite in a MgO–CaO–SiO_2_–P_2_O_5_–CaF_2_ glassy matrix.

However, fragility and poor mechanical strength of bioceramics have prevented their extensive applications as artificial bone replacement^23^. Incorporation of bioactive particles into the polymeric matrix to produce hybrids with enhanced mechanical and biological performance has been considered as a promising approach. The mass ratio and dispersion of the inorganic materials in the polymer matrix can be tailored in a way that the mechanical and physiological features of the composite meet the implant requirements. Most synthetic polymers possess high mechanical strength and flexibility while they suffer from low bioactivity in contrast to bioactive ceramics^24–26^. Among various synthetic polymers, polyethersulfone (PES) is regarded a suitable matrix due to its favorable features such as low price, high mechanical strength, resistance to hydrolysis, acidic and alkaline media^27^. Moradienayat et al.^28^ have prepared polysulfone/HA polymer nanocomposites by using solution spraying by airbrushing as a potential method for restoring bones. They have stated that mechanical properties of PSF/HA nanocomposites are highly conditioned by four highly interrelated factors: nanoparticle dispersion, nanoparticle–polymer interactions, and interphase and nanoparticle concentration. PES as a bioinert polymer can preserve its molecular structure in contact with radiations, which make it possible to be sterilized prior to in-vivo implementation^29^. On the other hand, structural consistency and low disintegration rate in physiological environment can retain the delivery of the integrated drug for a long time and prevent the burst release, which is inevitable when highly degradable polymers are used as carrier. Azadbakht et al.^30^ have also verified the cyto-compatibility of PES by culturing HepG2 cells.

Dexamethasone (DEX) has been recently studied as a water-insoluble anti-inflammatory drug, with immunosuppression capability that also stimulate osteogenic differentiation^31,32^. Whereas, the release of high quantity of DEX may imperil the viability of trabecular cells^33^. Hence, it is critical to reduce the therapeutic dosage to control the negative results related to the DEX exposure. Development of a system that manage the drug release pattern, while inhibit the local irritation can be a reliable strategy.

In order to develop a biodegradable drug carrier with proper bioactivity approximating ideal bone filler, PES/HA/WS/DEX composites were studied in the present research. The influence of the weight ratio of WS and HA on the microstructure, in-vitro bioactivity and cyto-compatibility, degradability and drug delivery behavior of the prepared composites were studied.

## Materials and methods

### Materials

Polyethersulfone (PES, Radel® A300) pellets were supplied by Amoco Chemicals. calcium nitrate tetrahydrate Ca(NO_3_)_2_.4H_2_O, diammonium phosphate (NH_4_)_2_HPO_4_, ammonium hydroxide (NH_4_OH) and sodium metasilicate nonahydrate Na_2_SiO_3_. 9H_2_O were supplied by Merck, Dexamethasone (DEX) (4 mg/mL) was purchased from an Iranian pharmaceutical manufacturing. All chemicals were of the analytical grade and used without purification.

### Synthesis of the wollastonite

In order to prepare WS powder, a simple hydrothermal procedure was followed, as previously described^34,35^. First, 0.5 M aqueous solutions of the analytical-grade Na_2_SiO_3_. 9H_2_O and Ca(NO_3_)_2_. 4H_2_O were prepared, respectively. The obtained solutions were mixed slowly under vigorous stirring at ambient temperature; this led to the formation of a white suspension. A Teflon-lined stainless-steel autoclave was used to heat the suspension up to 200 °C for 1 day. After the thermal treatment and cooling down the container, the solution was filtered and washed with distilled water, followed by ethanol. The final product was achieved upon drying the white powders at 100 °C for 1 day, followed by calcination at 1000 °C for 2 h in a furnace.

### Fabrication of PES/HA/WS composite specimens

Initially, 5 g of PES was dissolved in 50 mL of dimethylformamide (DMF) and a homogeneous solution was obtained. Then, different weight percentages (stated in Table 1) of the previously synthesized HA^36,37^ and also the prepared WS were included in the polymer solution, respectively. The whole procedure was conducted under vigorous stirring to avoid agglomeration, followed by ultrasonication for 1 h to disperse the particles uniformly. The final solutions were cast on a Petri dish and the composite specimens achieved via solvent evaporation technique. Afterwards, the composite samples were separated from the Petri dish, dried at 50 °C for 2 h and then at 110 °C for 5 h to remove any remained solvent..

**Table 1:** Reagents used for the fabrication of specimens.

Sample code	Reagents			
	PES (wt.%)	HA (wt.%)	WS (wt.%)	DEX (mg)
PES/HA/DEX	10	2	0	4
PES/HA/2 WS/DEX	10	2	2	4
PES/HA/4 WS/DEX	10	2	4	4
PES/2 WS/DEX	10	0	2	4
PES/DEX	10	0	0	4

### In-vitro drug loading and release study

DEX as a commonly used anti-inflammatory was chosen as the model to evaluate the drug release behavior. To prepare the drug loaded composites, DEX was incorporated into the composites following the same procedure as depicted in Section “Fabrication of PES/HA/WS composite specimens”. A series of drug carriers were fabricated based on the following procedure. 4 mg of DEX was added to each 10 mL of the composite solution and stirred for several hours to ensure the complete dispersion of drug before pouring in petri dishes.

The profile of the released drug from the composite samples was determined by using a UV–Vis spectrophotometry at λ = 241 nm over a period of 16 days. The prearranged quantity of samples was immersed in the phosphate buffered saline (PBS) of pH 7.4 at 37 °C and shaken gently. Each sample was placed in a separate vial, which was taken at certain time to analyze the solution by UV–Vis spectroscopy. The drug release study was performed in triplicate for each sample. Various concentrations of DEX were analyzed by UV–Vis spectroscopy to plot the calibration curve. The weight ratios of the utilized precursors to prepare the composite specimens were summarized in Table 1.

### Characterization

The X-ray diffractometer (PANalytical's X'Pert Pro, Netherland) was used to analyze the phase composition of the prepared specimens. The spectra were recorded in the 2θ of 10°–80° with CuKα (λ = 1.5406 Å) as the radiation source at a current of 30 mA and with an accelerating voltage of 40 kV. The composition of the present phases was identified by comparing the X-ray patterns with JCPDS (files no. 09–0432 and no. 43–1460) standards. The chemical structure of the prepared samples was investigated by using Fourier transform infrared (FT-IR) spectroscopy (Rayleigh, China) over the range of 4000 and 400 cm^-1^ at a resolution of 1 cm^-1^ using KBr pellets.

To study the surface morphology and elemental composition of the samples, field emission electron microscopy (FESEM) (Zeiss HV-300-Germany) associated with energy dispersive X-ray analyzer (EDX, Oxford AZtec1-England) was used. Prior to analysis, specimens were gold sputtered by applying a 20 mA current for 30–60 s to form a 5–10 nm coating layer.

### In-vitro bioactivity study

The in-vitro bioactivity of the obtained specimens was investigated via formation of a bone-like apatite layer on the surface after submersion in SBF that resembles the ion quantity of human blood plasma as expressed in Table 2 and also previously reported by Kokubo^38^. In summary, a predetermined amount of the specific reagents was dissolved in distilled water; Tris-buffer hydrochloric acid (HCl) was used to retain the pH around 7.4. The fabricated specimens were immersed in the SBF at 37 °C for 3 weeks, where the proportion of surface area (cm^2^) to solution volume (mL) was about 0.1; followed by removing the specimens from the SBF solution, rinsing with water, and finally drying in an oven prior to further characterization. The GI-XRD, ATR-FTIR, FESEM and EDX analysis were used to study the generated layer on the composites surface.

**Table 2:** The ions dosage in SBF compared with human blood plasma.

Types	Ion concentrations (mM)						
	Na^+^	K^+^	Mg^2+^	Ca^2+^	Cl^-^	HCO_3_^-^	HPO_4_^2-^
SBF	142	5	1.5	2.5	148.8	4.2	1
Blood Plasma	142	5	1.5	2.5	103	27	1

### In-vitro bioresorbability study

To study the stability of the produced specimens in physiological conditions, they were soaked in the PBS medium of pH 7.4 at 37 °C for 16 days. The released amount of the calcium ions in the buffer solution was measured by flame photometer (Clements, Germany).

### In-vitro biocompatibility study

Cytotoxicity of the obtained specimens was evaluated via MTT (3-(4,5- dimethylthiazol-2-yl)-2,5-diphenyl tetrazolium bromide) assay by employing human fibroblasts (A-431 cell line ) provided by the cell bank of Iran Pasteur Institute. The cells viability depicts the composites biocompatibility^39^.

In brief, 10^4^ fibroblast/well were seeded into a 96-well plate containing DMEM medium supplemented with 10% fetal calf serum, incubated at 37 °C with 100% relative humidity and 5% CO_2_ for several hours, followed by treating with various concentrations (25, 50 and 100 μg/ml) of the samples. The empty wells were considered as the control groups in the assay. After 1 day of incubation, the supernatant was taken out of the wells. Prior to subjecting the specimens to MTT test, an inverted microscope (CKX, Olympus) was utilized to observe the density of cells in each well and capture images at 40× . Then, 0.5% MTT solution was added to the plates and incubated at 37 °C for 4 h. After aspirating the culture medium, 0.2 ml of DMSO was poured into each well and the UV–Vis spectrophotometer was used to read the absorbance of the final solution at 492 nm. Each test was carried out in triplicate and the viable cells were quantified based on the absorbance values. The measured values for control were considered as 100% to determine the cell viability percentage.

### Statistical analysis

All data were expressed as mean ± SD. Two-way analysis of variance (ANOVA) was performed and P < 0.0001 was considered to indicate statistical significance.

## Results

### Characterization of the prepared specimens

**Figure 1:**
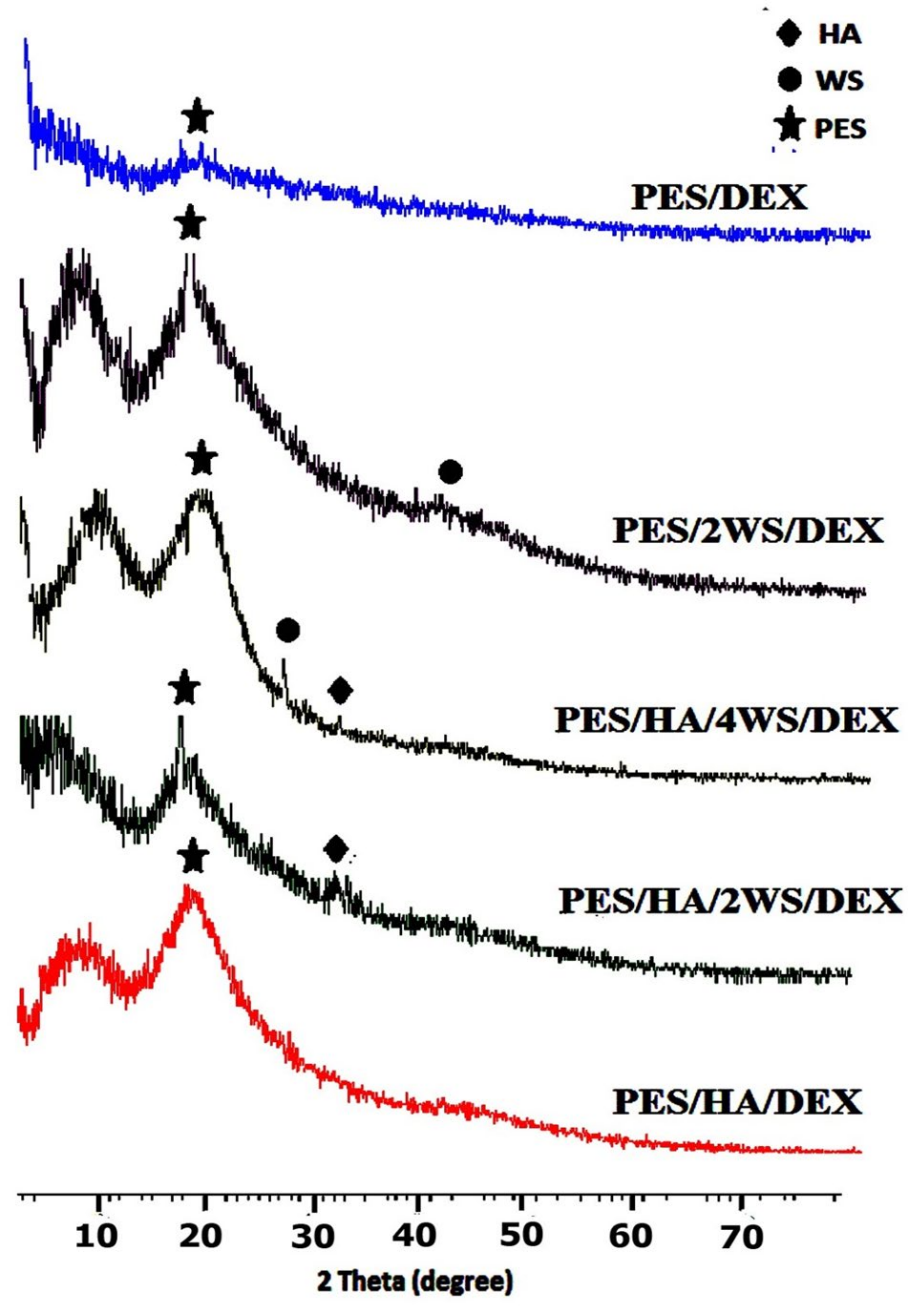
GIXRD pattern of the prepared films prior to soaking in SBF.

Figure 1 shows the XRD pattern of the prepared thin film specimens. The broad peak distinguished around 2θ = 18° for all samples was ascribed to the amorphous PES; this was in agreement with the previously reported data by Nair et al.^40^, where a 25µm thick PES foil represented an amorphous peak at around 2θ = 18°. Guan et al.^41^ and Kumar et al.^42^ also observed a peak at 2θ = 18.78° and 19.9° for a PES film, respectively. The appeared peak around 2θ = 31.8° in the pattern of PES/HA/2 WS/DEX and PES/HA/4 WS/DEX, was related to the (211) plane in HA that indicated the presence of HA in the composites, in accordance with the standard value of HA (JCPDS file no. 09–0432)^36^. Meanwhile, the observed peaks at 2θ = 27.1° for PES/HA/4 WS/DEX and 2θ = 41.5° in PES/2 WS/DEX patterns, respectively, could be corresponded to the (202) and (040) planes in WS, compared with the standards (JCPDS file no. 09–0432)^43^.

**Figure 2:**
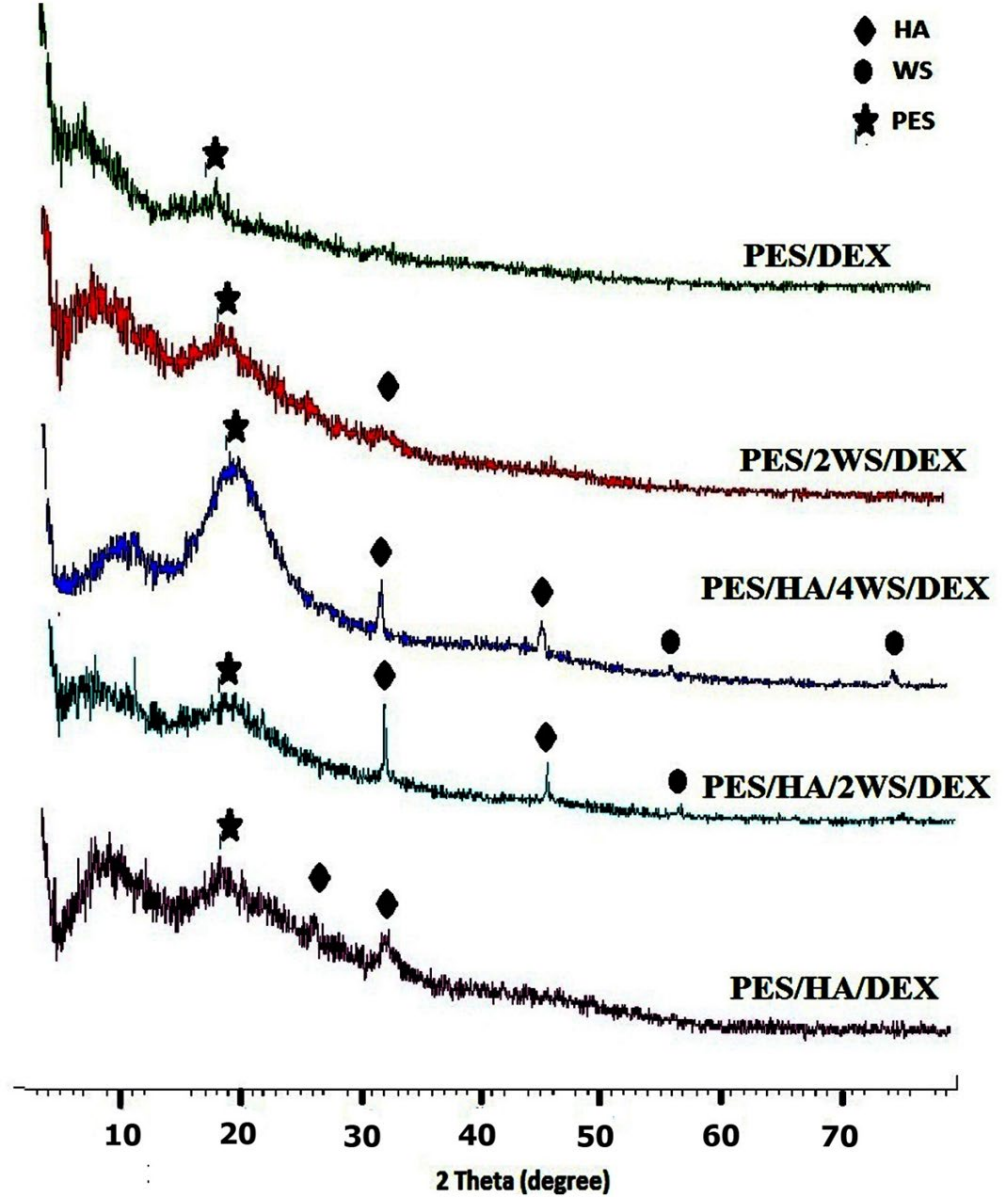
GIXRD pattern of the prepared films after soaking in SBF up to 21 days.

Upon placing the thin films in SBF for 3 weeks, new peaks were observed in the X-ray diffraction patterns of all composites as shown in Fig. 2. The appeared peaks at 2θ = 25.7° and 31.6° in the PES/HA/DEX, 2θ = 31.6° and 46.4° in the PES/HA/2WS/DEX and PES/HA/4 WS/DEX, respectively, and also at 2θ = 31.6° in the PES/2 WS/DEX patterns, suggested the precipitation of an apatite layer on the specimens surface^44^. Whereas, low bioactivity of PES could not incite the precipitation of calcium and phosphate ions on the surface to form an apatite layer and no characteristic peaks were observed for PES/DEX sample prior to and upon immersion in SBF. Moreover, the detected peaks around 2θ = 56.4° and 75° with low intensities were corresponded to WS particles in the composites^45^.

**Figure 3:**
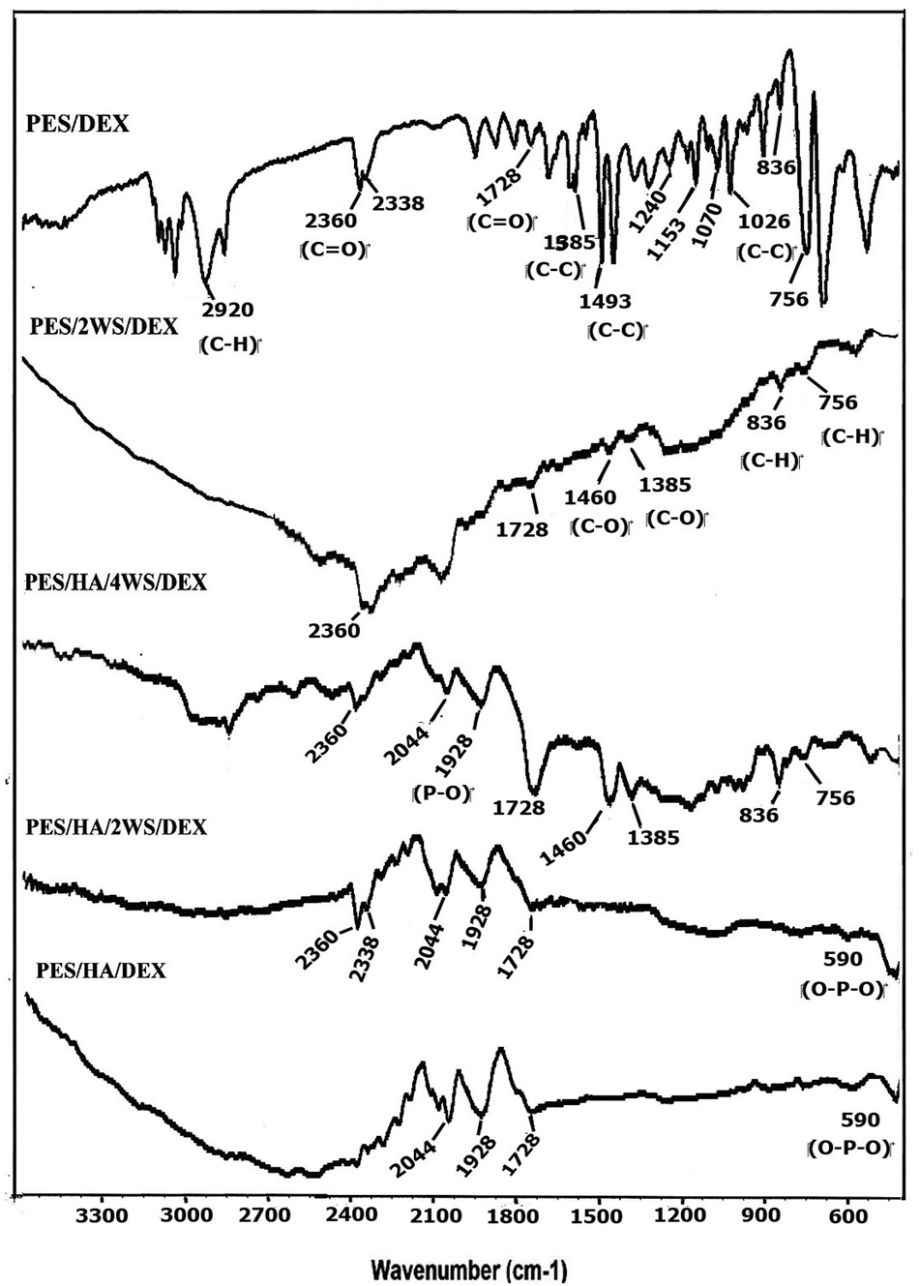
ATR-FTIR spectra of the prepared films before immersion in SBF.

The ATR-FTIR spectra of the prepared samples were depicted in Fig. 3. The observed sharp peaks at 1026, 1493 and 1585 cm^-1^ corresponded to the (C–C) vibration^46^, at 2920, 836 and 756 cm^-1^ ascribed to the (C–H) vibration^47^, at 1240 cm^-1^ related to the (ether) group^48^, at 1153 and 2338 cm^-1^ due to the (sulfone groups)^48,49^, at 1728 and 2360 cm^-1^ due to the (C=O) vibration in DEX^50^, and at 1070 cm^-1^ corresponded to the (C–O–C) vibration, in the PES and DEX chemical structure^37,51^. Carballo-Meilan et al.^52^ ascribed the observed band at 1928 cm^-1^ to the 1st overtone P–OH and C=O stretch. Moreover, the band at wavenumber 2044 cm^-1^ could be related to the DMF used as solvent in the experiments.

Generally, the stretching vibrations of Si–O in WS can be found around 900–1100 cm^-1^^45,53^; this band was overlapped with the P-O asymmetric absorption band in the HA, which is usually appeared in the range of 950–1100 cm^-1^^54^. The appeared bands around 590 cm^-1^ in the spectra of PES/HA/DEX and PES/HA/2 WS/DEX could be attributed to the O–P–O vibration that came from the PO_4_^–3^ group in HA crystals, and around 1385 cm^-1^ corresponded with the stretching mode of the carbonate^55^.The C–O band in the carboxylate groups was detected at around 1460 cm^-1^ in the FTIR spectra^56^.

**Figure 4:**
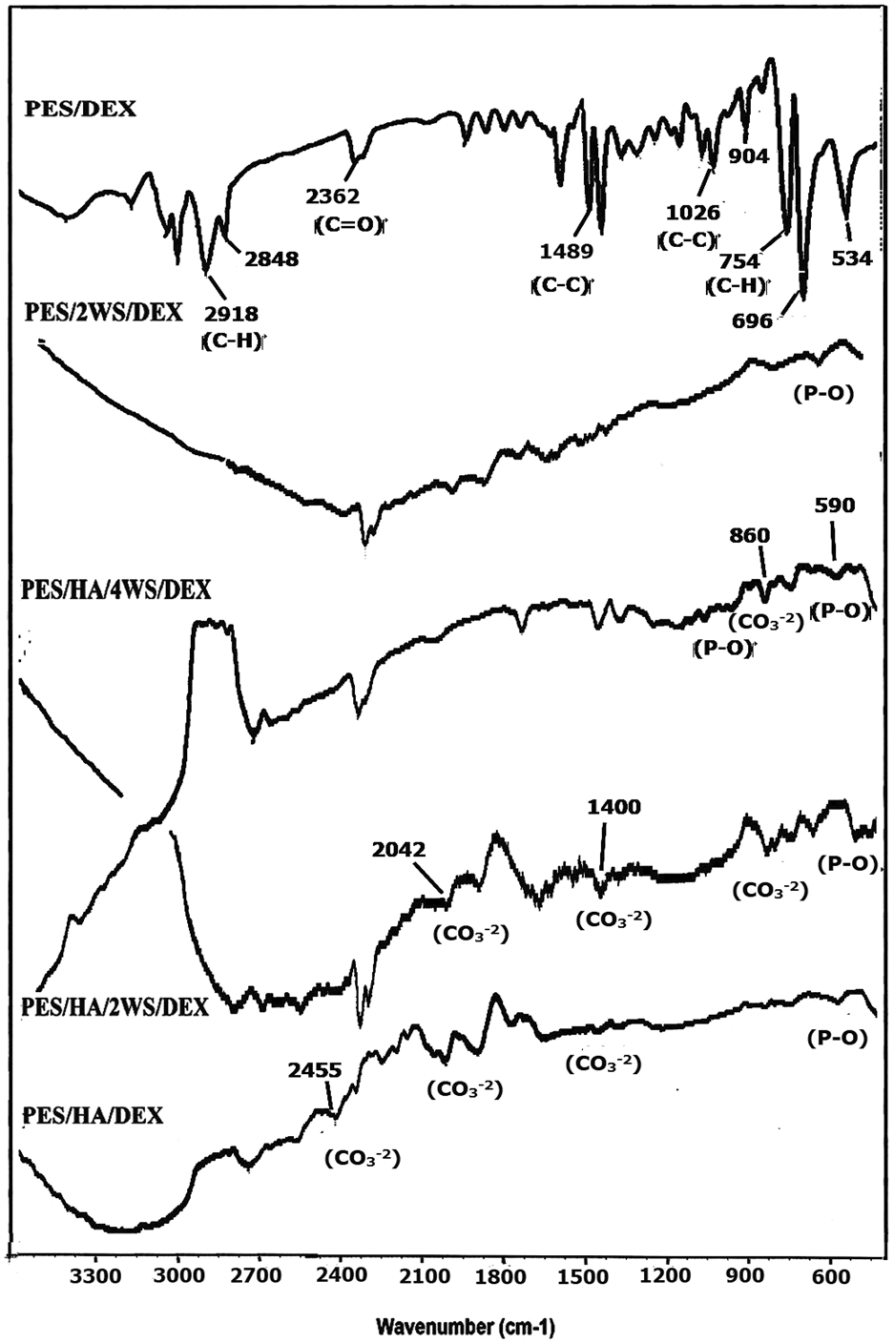
ATR-FTIR spectra of the prepared films upon immersion in SBF for 21 days.

Figure 4 shows the FTIR spectra of the prepared materials upon immersion in SBF. The absorption peaks around 590–700 and 980 cm^-1^ were due to the PO_4_^–3^ groups^44^. The absorption bands observed at 860, 1400, 1470, 2042 and 2455 cm^-1^ were ascribed to the carbonate groups^55,57^.

**Figure 5:**
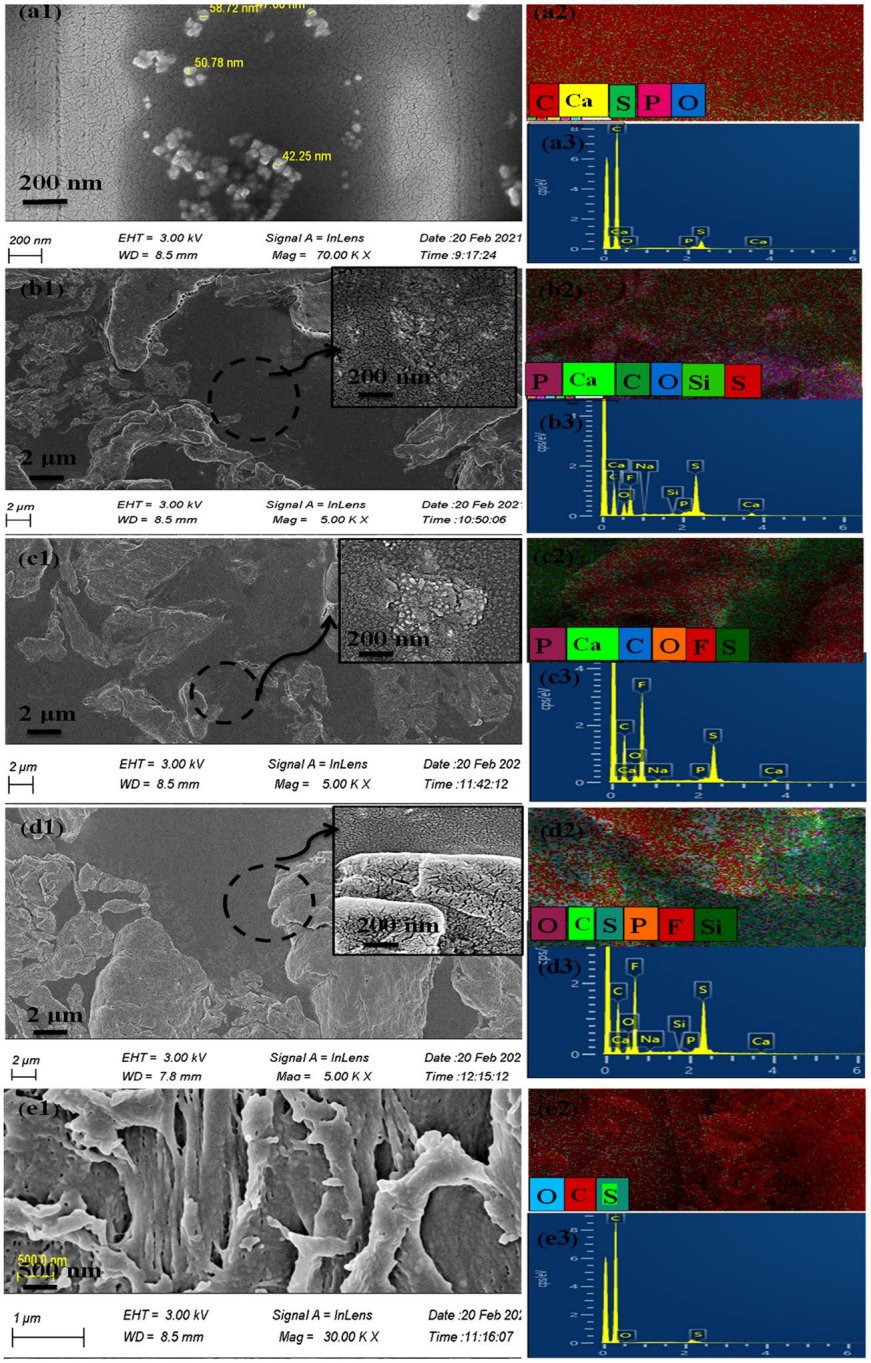
FESEM image and magnified view accompanied by elemental mapping and EDX data for the obtained samples of (**a**) PES/HA/DEX, (**b**) PES/HA/2WS/DEX, (**c**) PES/HA/4WS/DEX, (**d**) PES/2WS/DEX and (**e**) PES/DEX before immersion in SBF.

Figure 5 and Table 3 exhibited the FESEM images, EDX and elemental mapping of the prepared films. Heterogeneous distribution of the HA nanoparticles could be clearly observed in the Fig. 5a1, with no agglomeration. In the Figure 5b1 and c1, the presence of WS plate-like particles was clear, where the inserted high-magnification images disclosed the agglomerated HA nanoparticles. Figure 5e1 showed the polymer chains with no trace of inorganic particles. The EDX spectra accompanied by elemental mapping data were shown in Fig. 5a3–e3 and a2–e2, respectively, to recognize the elements and their spatial distribution throughout the films.

**Table 3:** Elemental composition of the prepared specimens prior to immersion in SBF.

Sample ID	Element (Atomic %)								
	Ca	P	O	Na	C	S	F	Si	Ca/P ratio
PES/HA/DEX	0.03	0.02	0.44	0	98.52	0.99	0	0	1.5
PES/HA/2 WS/DEX	0.42	0.27	12.77	0.2	63.10	5.1	18.06	0.08	1.55
PES/HA/4 WS/DEX	0.21	0.14	10.34	0.21	55.46	3.18	30.47	0	1.5
PES/2 WS/DEX	0.07	0.09	11.35	0.18	58.79	4.01	25.35	0.1	0.78
PES/DEX	0	0	1.05	0	98.78	0.17	0	0	0

**Figure 6:**
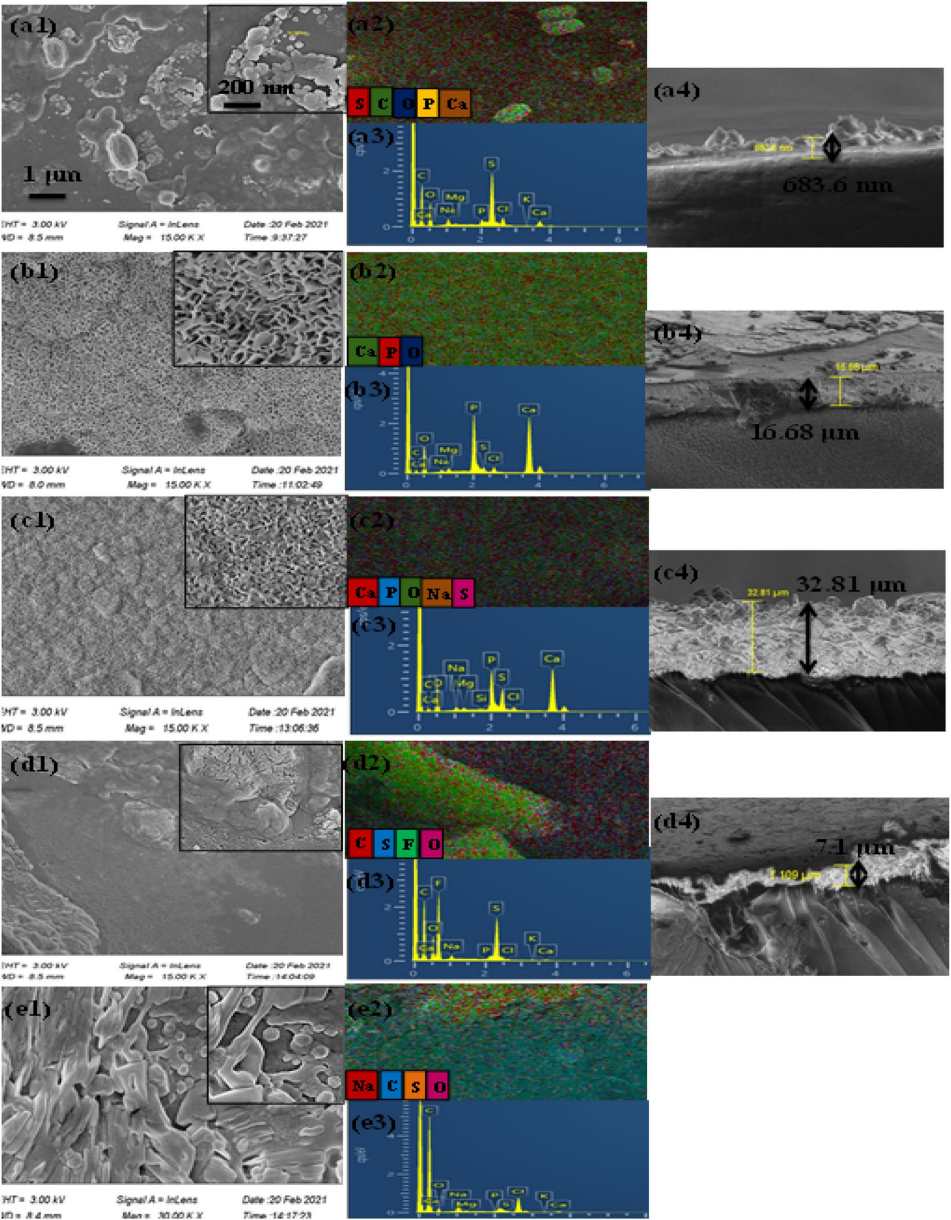
FESEM image and magnified view accompanied by elemental mapping, EDX analysis and cross-section view for the prepared samples of (**a**) PES/HA/DEX, (**b**) PES/HA/2WS/DEX, (**c**) PES/HA/4WS/DEX, (**d**) PES/2WS/DEX and (**e**) PES/DEX after immersion in SBF for 21 days.

The FESEM micrographs of the specimens placed in SBF for 3 weeks, EDX and elemental mapping were revealed in Fig. 6 and Table 4. Some small colonies of the precipitated apatite could be observed in Fig. 6a1, whereas the surface of the samples in Fig. 6b1 and c1 was completely covered by an apatite layer. No sign of precipitated apatite nuclei was detected on the surface of PES film in Fig. 6e1.

**Table 4:** Elemental composition of the prepared samples upon soaking in SBF.

Sample ID	Element (Atomic %)								
	Ca	P	O	Na	C	S	Cl	Mg	Ca/P ratio
PES/HA/DEX	0.55	0.37	19.21	0.59	74.38	4.15	0.65	0.06	1.49
PES/HA/2 WS/DEX	15.94	10.61	55.71	0.71	14.27	0.96	1.06	0.76	1.5
PES/HA/4 WS/DEX	11.91	7.35	61.07	1.00	12.79	4.63	0.74	0.46	1.62
PES/2 WS/DEX	0.03	0.03	12.37	0.35	65.17	2.54	0.06	0.1	1
PES/DEX	0.11	0.04	4.42	1.06	93.00	0.06	1.23	0.05	2.7

The cross-section view of the films in Fig. 6a4–e4, obviously showed the newly formed layer of calcium-phosphate, where the thickness varied depending on the composition of the samples.

### In-vitro bioresorbability study

**Figure 7:**
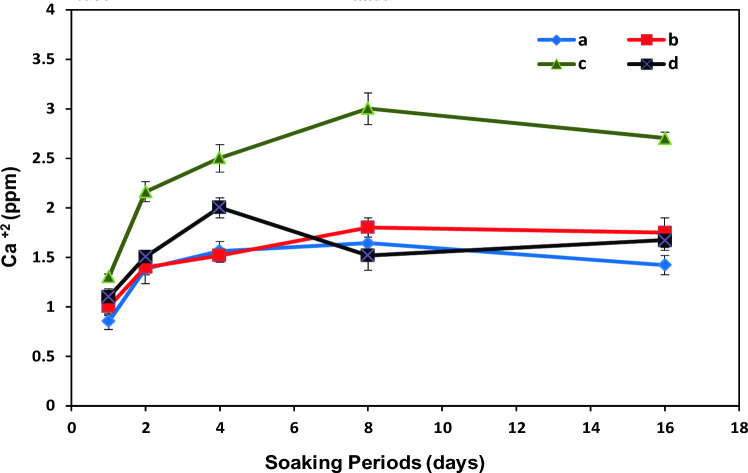
Variations of Ca^2+^ strength in buffer medium with soaking periods for (a) PES/HA/DEX, (b) PES/HA/2WS/DEX, (c) PES/HA/4WS/DEX and (d) PES/2WS/DEX.

The in-vitro resorption rate of the prepared composite films was investigated quantitatively in the PBS solution at pH 7.4 and 37 °C for 16 days, as depicted in Fig. 7. The variation of the Ca^2+^ ionic strength of the PBS medium was measured as an indication of the samples bioresorbability. The release rate of the Ca^2+^ ions was rapid during the early stage of immersion, which followed by a relatively unvarying trend as the soaking time increased. Most of the inorganic particles were imbedded within the polymeric substrate and were not in direct contact with PBS to get dissolved and release calcium ions, whereas some available HA and WS particles on the composites surface could react with the buffer solution and release high amount of Ca^2+^ upon immersion. Hence, it could be concluded that the fabricated composites were stable in physiological fluids for a long time. As stated by Salmani et al.^11^ the released calcium ions from the material after immersion in the solution could result in the absorption of H + and formation of silanol groups on surface of the porous. The following released ions resulted in negative charge on the material surface and therefore, the positively charged calcium ions was adsorbed to the surface. Finally, the changes in calcium ion release and absorption rate resulted in apatite formation on the material surface. So, the concentration of calcium ions in the solution did not change dramatically. Similarly, Foroutan et al.^58^ have prepared bone scaffolds based on alginate/wollastonite/grapheme nanosheets and indicated that presence of 2 wt% grapheme nanosheets could improve the chemical stability of the composite until 4 days, where the quantity of calcium and silicon ions started to goes up in the SBF solution.

### Drug release profile

**Figure 8:**
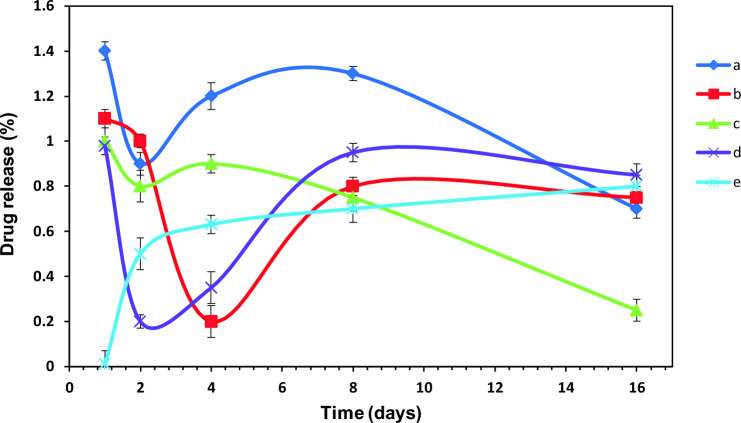
Drug release profiles for (a) PES/HA/DEX, (b) PES/HA/2WS/DEX, (c) PES/HA/4WS/DEX, (d) PES/2WS/DEX and (e) PES/DEX.

The drug release patterns for the prepared specimens were presented in Fig. 8. Evidently, all samples displayed a similar continuous release profile, upon the beginning the substrate degradation after 3–4 days of soaking in PBS. The characteristic burst release observed in the first day was related to the liberation of the available drug on the films surface as well as the unbound drugs^59^, which was followed by a dramatic decrease in the detected amount of the released drug; this implied that most of the drug was retained within the polymer matrix. Afterwards, the extended release time verified the dependence of the released drug to the substrate degradation. Low percentage of the released drug observed for all samples at the end of 16 days, was attributed to the excellent hydrolytic stability of ether bonds, as claimed by Sata et al.^60^.

The main difference between this graph and other common drug release graphs was that the cumulative release amount was not shown and the released amount was exactly the amount of drug that was released from the composite during a certain period of time. It means that, the released drug that was previously recorded and shown in the graph was subtracted from the total amount of released drug recorded that moment. Therefore, the quantity of drug in the solution was very high on the first day, which indicated the release of drug molecules that were attached to the surface of the composite. But after one day, a sharp drop was seen in the graph. It meant that the amount of drug released in the solution during 2 days minus the amount of released drug recorded before was very small. Therefore, it could be said that the composite was almost stable with no degradation. But after the passage of time and with the beginning of the destruction of the composite, the amount of drug released from the samples increased slightly and almost a constant amount of the drug was released every day, which suggested the slow and regular degradation of the substrate.

### Cytotoxicity study

**Figure 9:**
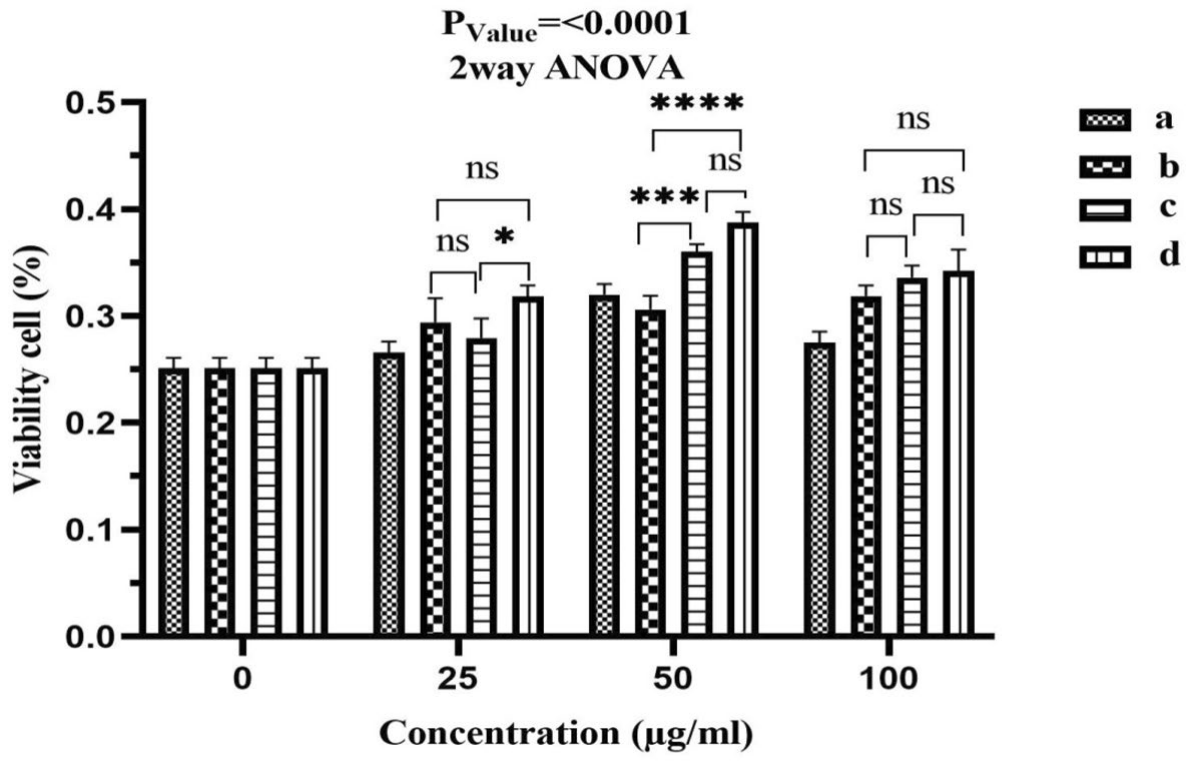
MTT assay results for the viability of fibroblast cells in contact with various concentrations (25, 50 and 100 μg/mL) of the (a) PES/HA/DEX, (b) PES/HA/2WS/DEX, (c) PES/HA/4WS/DEX and (d) PES/2WS/DEX films for 24 h. Results of three independent replicates are shown as means ± SD. Statistic testing was performed using a two-way ANOVA. ****p < 0.0001, ns = non-significant.

**Figure 10:**
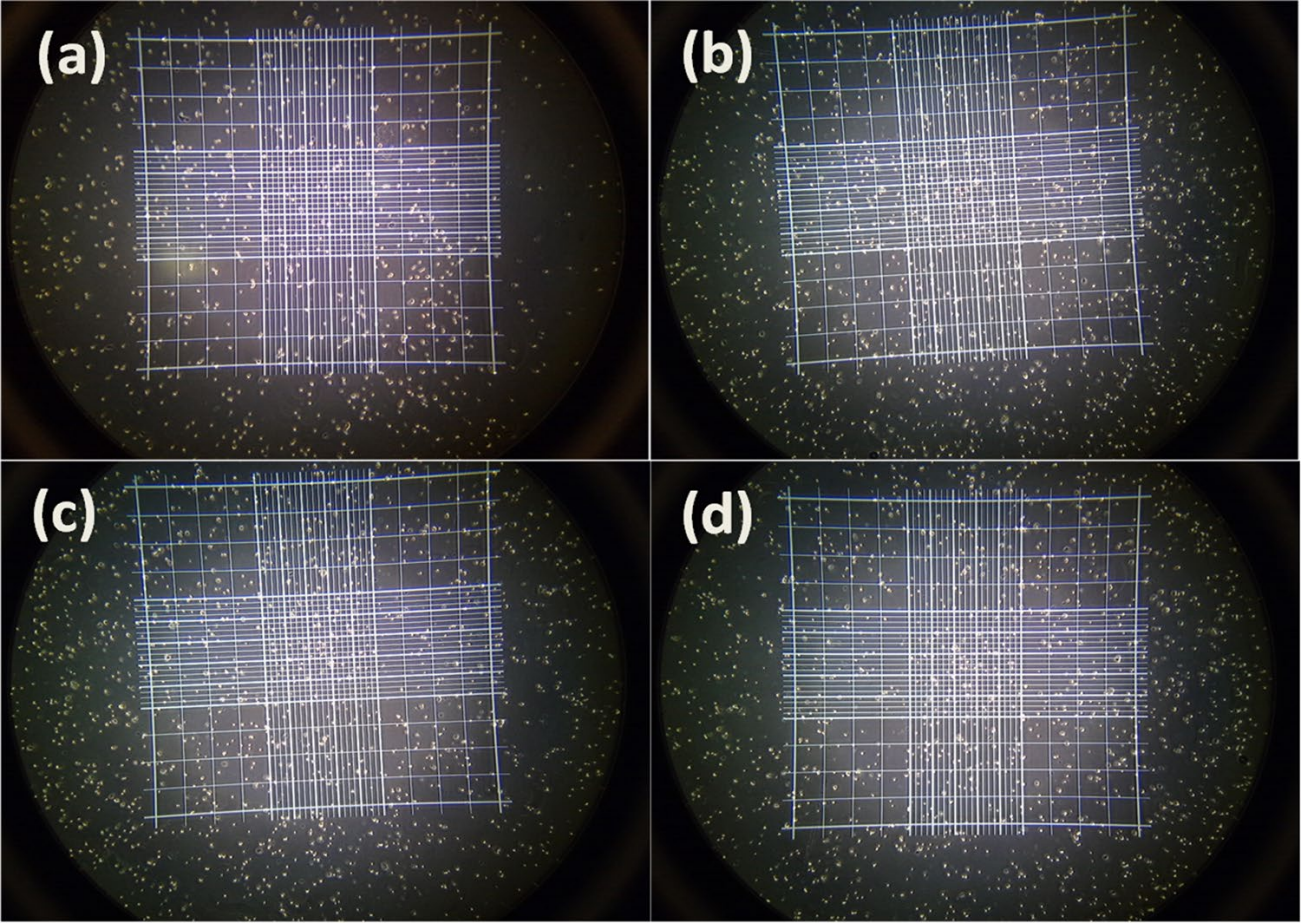
Optical microscopy images of trypan blue stained fibroblast cells incubated with 100 μg/mL of the (**a**) PES/HA/DEX, (**b**) PES/HA/2WS/DEX, (**c**) PES/HA/4WS/DEX and (**d**) PES/2WS/DEX films for 24 h.

The materials used in tissue engineering should represent the least cytotoxicity. In this regard, the potential cytotoxicity of the obtained samples was estimated via MTT method, where different concentrations of the extracted liquid of the specimens came in contact with the fibroblast cells as shown in Figs. 9 and 10.

None of the fabricated films (PES/HA/DEX, PES/HA/2WS/DEX, PES/HA/4WS/DEX and PES/2WS/DEX) could limit the proliferation of fibroblast cells. In addition, higher concentration of the WS could increase the cyto-compatibility of PES/HA/2WS/DEX hybrid films, implied the significance of the WS quantity on the cells viability and proliferation. Supposedly, the observed tremendous improvement in the proliferation of fibroblasts in contact with higher concentration (50 and 100 μg/mL) of the extracted liquid could be attributed to the favorable cell signaling of these compounds. All the films displayed more than 100% cell viability. Basically, the cell/material interactions and the potential of the cells to attach, grow and proliferate, relied not only on the material surface features but also on its chemical compositions^61,62^. Any variation in the chemical compositions can influence the cell responses, since determine the amount of the liberated ions from the material. It was reported that the ionic products came from the dissolved material, especially Si and Ca from bioactive glasses are able to motivate the proliferation of osteoblasts and expression of genes^63–65^. As also investigated in this research, the level of the released Ca from PES/HA/4WS/DEX sample was high than others. On the other hand, MTT tests indicated that higher concentrations of materials could stimulate the fibroblast cells growth and proliferation, significantly. So, it could be concluded that the continuous dissolution of HA and WS can create a calcium and silicon-rich medium, which was believed to be responsible for the motivation of the cells growth and proliferation, particularly after incubation with higher concentrations of the samples. Generally, the initial interactions between the surface of the implanted materials and the surrounding cells are believed to be one of the determining issues in therapeutic achievement of biomaterials. Further detailed and systematic cell studies are, however, essential in the future.

An optical microscope was employed to monitor the morphology of the cells upon 24 h incubation with 100 μg/mL concentration of the specimens, as depicted in Fig. 10. The cells attachment to the flasks, proliferation and colonies formation was obviously observed. Compared to the PES/HA/DEX, and PES/HA/2WS/DEX films, the cells in contact with the PES/HA/4WS/DEX and PES/2WS/DEX films could form denser colonies, especially after incubation with high dosage (100 μg/mL) of the samples, implying the significance of WS in improving the biocompatibility of the composites.

## Discussion

The X-ray patterns as expressed in Fig. 2 of the films upon immersion in SBF for 21 days demonstrated that the diffraction peaks related to HA were more distinguished than the WS peaks, implying that almost the entire surface was covered by the crystalline HA (as indexed in the JCPDS file no 01-072-1243). However, no peak appeared in the PES/DEX pattern of Fig. 2, revealing the very low bioactivity of the PES film.

Emergence of new absorption bands at 1400 and 1470 cm^-1^ in the spectra of Fig. 4 (PES/HA/DEX, PES/HA/2WS/DEX, PES/HA/4WS/DEX) was related to the carbonate groups, revealing that the carbonates have been substituted in phosphate sites in the precipitated apatite structure^66^. The formation of CO_3_^–2^ ions might be attributed to the reaction between hydroxyl groups of the solution and CO_2_ of the air. These spectra strengthened the idea that the bone-like hydroxycarbonate apatite covered the surface of the specimens contained HA and WS. Low intensity of the absorption bands in the Fig. 4 (PES/2WS/DEX), especially at 860, 1470 cm^-1^ happened due to the difficulty in the detection of carbonate groups, which showed the low quantity of the nucleated carbonate apatite on the surface.

Figure 5a2–e2 and a3–e3 disclosed the presence of C and S, which was related to the PES polymeric framework. Identification of F element in the Fig. 5b3–d3 showed the existence of DEX on the films surface. The Ca/P ratio was found to be around 1.5, but showing differently (0.78) for PES/2 WS/DEX specimen (Table 3). The observed deviation from 1.67 (for HA) could be related to the existence of phosphate ions in the DEX ampule purchased from a local pharmaceutical manufacturer that affected the ratio of Ca to P, as measured by EDX.

Kokubo et al. has proposed that formation of an apatite layer on a material surface in SBF is a feasible way to forecast its in-vivo bioactivity and the aptitude to form interfacial bonds with living bone^67^, which has not been found in non-bioactive materials^10^. Several reports have discussed the potential of incorporating bioactive ceramics into the polymeric matrix to create bioactive composites for filling bone defects^68,69^. The present study tried to combine HA and WS as degradable and bioactive components with the PES to obtain novel bioactive composites. The in-vitro bioactivity was investigated via formation of an apatite layer on the surface after placing in SBF. Moreover, DEX was also incorporated into the composites to study the release behavior.

The main elements detected by elemental mapping and EDX analysis were S, C, O, P and Ca, as represented in Fig. 6a2 and a3, where the Ca to P ratio was around 1.49, indicating the precipitation of calcium-deficient apatite particles on the surface. The morphology of the newly created apatite layer was entirely distinct from the apatite nanoparticles in the composite, which was obviously observed in the magnified FESEM images; enabling the observation of the surface variations before and after immersion in the SBF. The formation of a continuous layer of apatite on the surface of the composites in the Fig. 6b1 and c1 depicted the high degree of in-vitro bioactivity of the prepared composites, where the Ca/P ratios were 1.5 and 1.62, respectively (Table 4), which were closely similar to the 1.67 Ca/P ratio of bone-like apatite. Based on the EDX spectrum and elemental map data, low quantity of carbon element was detected for sample shown in Fig. 6b and c; this could be related to the carbonate groups in the apatite structure, as also indicated in the FTIR results and means that the PES/WS/HA/DEX composites were completely covered by a bone-like carbonate- apatite layer, verifying the potential capability of the obtained composites to be used as bone filler. Moreover, the observation of some trace elements, like Mg and Na in the Fig. 6b3 and c3, further endorsed the similarity of the formed apatite with the biological apatite composition. The thickness (32.8 μm) of the dense and continuous apatite layer nucleated on the surface of composite film in Fig. 6c4 was higher than other samples due to the remarkable bioactivity of the PES/HA/4WS/DEX composite.

Supporting evidence of the chemical elements obtained from EDX results indicated that the PES/WS film shown in Fig. 6d2 and d3 represented a very low degree of bioactivity where identification of calcium or phosphorous ions was difficult. Observation of a thin layer in Fig. 6d4 revealed the precipitation of calcium phosphate crystals on some areas of the surface, which was in accordance with the high amount of C and S, as identified in the Fig. 6d2 and d3. On the contrary, the formation of the mineral phase on the PES surface shown in Fig. 6e1 did not happen; this was supported by the EDX data, which indicated the presence of only high quantity of C that was related to the polymer backbone.

Consequently, the obtained results verified that the PES/HA/4WS/DEX composite with WS content 4wt% exhibited significantly improved in-vitro bioactivity. A higher amount of WS compared to the PES/HA/2WS/DEX nanocomposite film could be the probable reason for this activity.

Precipitation of an apatite layer on the surface of most of the polymers as well as other types of materials has not been observed upon exposure to SBF. On the other hand, the deposition of calcium phosphate films on polymers through a ‘‘bio-mimetic’’ process has been studied^70,71^. In this case, many systems, when introduced into an over concentrated solution of calcium and phosphate ions, can form an apatite layer. It has been indicated that presence of hydrophilic groups on the polymers, metastable concentrations of calcium and phosphorous in solution, and in some cases, silicate ions in solution are required for the deposition of apatite on polymers in the so called ‘‘bio-mimetic’’ process^72^.

In the case of bioactive glass-polyethersulfone composites, it seems that silicate ions in solution play a key role in the precipitation of apatite. Ether bonds in polyethersulfone are surrounded by bulky phenol groups that can limit the adsorption of species containing silanol groups. Otherwise, silicate ions in solution can increase the ionic strength of the solution, reducing the barrier for apatite precipitation. The increase in ionic strength of the solution, corresponding to the calcium and phosphate ions (leached from the bioactive glass and HA particles) can lead to the precipitation of apatite on the surface of polyethersulfone exposed to SBF.

Hench et al.^73^ has suggested the mechanism of the apatite formation on the surface of bioactive compounds. Bioglasses can exchange calcium ions with hydrogen H_3_O^-^ in SBF and leave Si–OH groups behind, which had been claimed to have catalytic effect and play as promising sites for the nucleation of apatite crystals^14,74^. So, it can be proposed that the amount of the attainable Si–OH functional groups on the biomaterial surface has close relation with the apatite precipitation, which can be considered as the main reason for the observed discrepancy between the bioactivities of specimens, especially for PES/HA/4 WS/DEX composite with the highest WS content.

Degradability is also an important property of the third-generation biomaterials^75^. The obtained results in the Fig. 7 showed that the amount of the released Ca^+2^ from the PES/HA/4WS/DEX composite film was more than other samples; this was in agreement with the reported data by Lin et al.^76^ which suggested that the degradation of HA/WS composite ceramic increase with the increase in the WS content. Therefore, regarding the very low degree of PES degradation rate, the degradability of the composite films can be controlled by adjusting the primary WS content. However, the drug release profiles in the Fig. 8 revealed that the DEX-loaded samples have a similar release behavior, which could be ascribed to the good interaction between DEX and polymeric matrix and also the low degradation rate of PES.

## Conclusions

The synthesized HA and WS powders were blended with a PES matrix to prepare new organic–inorganic bioactive composite films, which contained DEX as an anti-inflammatory drug model to analyze the release behavior. The obtained results expressed that the highly biocompatible films contained both HA and WS had the aptitude to induce the formation of apatite after immersion in SBF, where the quantity of CaSiO_3_ in the composite films was the controlling item in the in-vitro apatite formation. The degree of bioactivity could not dramatically impact the degradation behavior of the composite samples and a slow and continues drug release pattern was observed.

Based on the concentration of calcium ions in the buffer solution and the released drug, it could be concluded that the obtained composites had very low degradation rate. Compared with other bioactive polymeric composites which usually employ degradable polymers such as gelatin, alginate, polyesters and etc. that loose their integrity in short time, the fabricated composites in this study could represent high values of bioactivity and moreover, preserve their structure for long time in biological conditions. Which make them potential candidate as permanent prostheses in bones or joints or as bone substitutes for long time.

## Data Availability

All data used during the study are available from the corresponding author by request.
